# Adverse reaction to Coartem (artemether/lumefantrine) resulting in oculogyric crisis

**DOI:** 10.1186/s40902-021-00300-y

**Published:** 2021-05-17

**Authors:** Emmanuel Kofi Amponsah, Buyanbileg Sodnom-Ish, Aaron Sowah Anyetei-Anum, Paul Frimpong, Soung Min Kim

**Affiliations:** 1 Oral and Maxillofacial Microvascular Reconstruction LAB, Ghana Health Service, Brong Ahafo Regional Hospital, P.O. Box 27, Sunyani, Brong Ahafo Ghana; 2 Department of Oral and Maxillofacial Surgery, Dental Research Institute, School of Dentistry, Seoul National University, 101 Daehak-ro, Jongno-gu, Seoul, 03080 Korea

**Keywords:** Antimalarial, Artemether-lumefantrine, Rapid diagnostic test, Oculogyric crisis, Dystonic reactions

## Abstract

**Background:**

Artemether/lumefantrine (AL), sold under the brand name Coartem, is the most common artemisinin-based combination therapy for the treatment of malaria. Drug-induced oculogyric crisis is a neurological disorder characterized by frequent upward deviations of the eye. In the literature, no cases of Coartem-induced oculogyric crisis have been reported in Ghana.

**Case presentation:**

A 19-year-old male patient, who presented fever measuring 37.9 °C, general body pains, and weakness was prescribed with antimalarial therapy artemether/lumefantrine, Coartem®, from a local pharmacy. Just after initiation of treatment, the patient complained of double vision, involuntary upward eye deviation, and inability to close both eyes. The patient was diagnosed with Coartem-induced oculogyric crisis and was treated with the cessation of the causing agent and intramuscular injection of promethazine hydrochloride.

**Conclusions:**

When a patient exhibits a neurological disorder, such as oculogyric crisis, with normal conscious state and normal vital signs, special attention should be given to obtaining a history of recently administered medications. Clinicians should recognize adverse reactions to drugs based on a thorough patient history and examination. The goal of this report was to present Coartem-induced oculogyric crisis.

## Background

Artemether/lumefantrine (AL), sold under the brand name Coartem, is the most common artemisinin-based combination therapy recommended by the World Health Organization. Since *Plasmodium falciparum* became resistant to chloroquine, AL has been used to treat malaria. It is not typically used to prevent malaria and is administered orally. Common side effects include muscle and joint pain, fever, loss of appetite, and headache. One serious side effect is prolongation of the QT interval. While not well studied, the use of AL in pregnancy appears safe [1]. The dosage of AL does not need to be changed in patients with mild or moderate kidney or liver problems. AL is on the World Health Organization’s Model List of Essential Medicines [2] and is not available as a generic medication [3].

## Case presentation

A 19-year-old male patient reported to the Oral & Maxillofacial Department of Brong Ahafo Regional Hospital in Sunyani, Ghana, with involuntary, spontaneous, conjugated, and rhythmic upward eye deviation and inability to close both eyes. Two days prior to presentation at our facility, he visited a pharmacy with complaints of fever measuring 37.9 °C, general body pains, and weakness. A rapid diagnostic test (RDT) conducted at the pharmacy was positive for *Plasmodium falciparum*. Therefore, the patient received antimalarial therapy artemether/lumefantrine, Coartem® (Novartis Pharma AG, Basel, Switzerland), at a dose of 80/480 mg and to be taken after 8 h on day one and twice daily thereafter for 2 days.

**Fig. 1:**
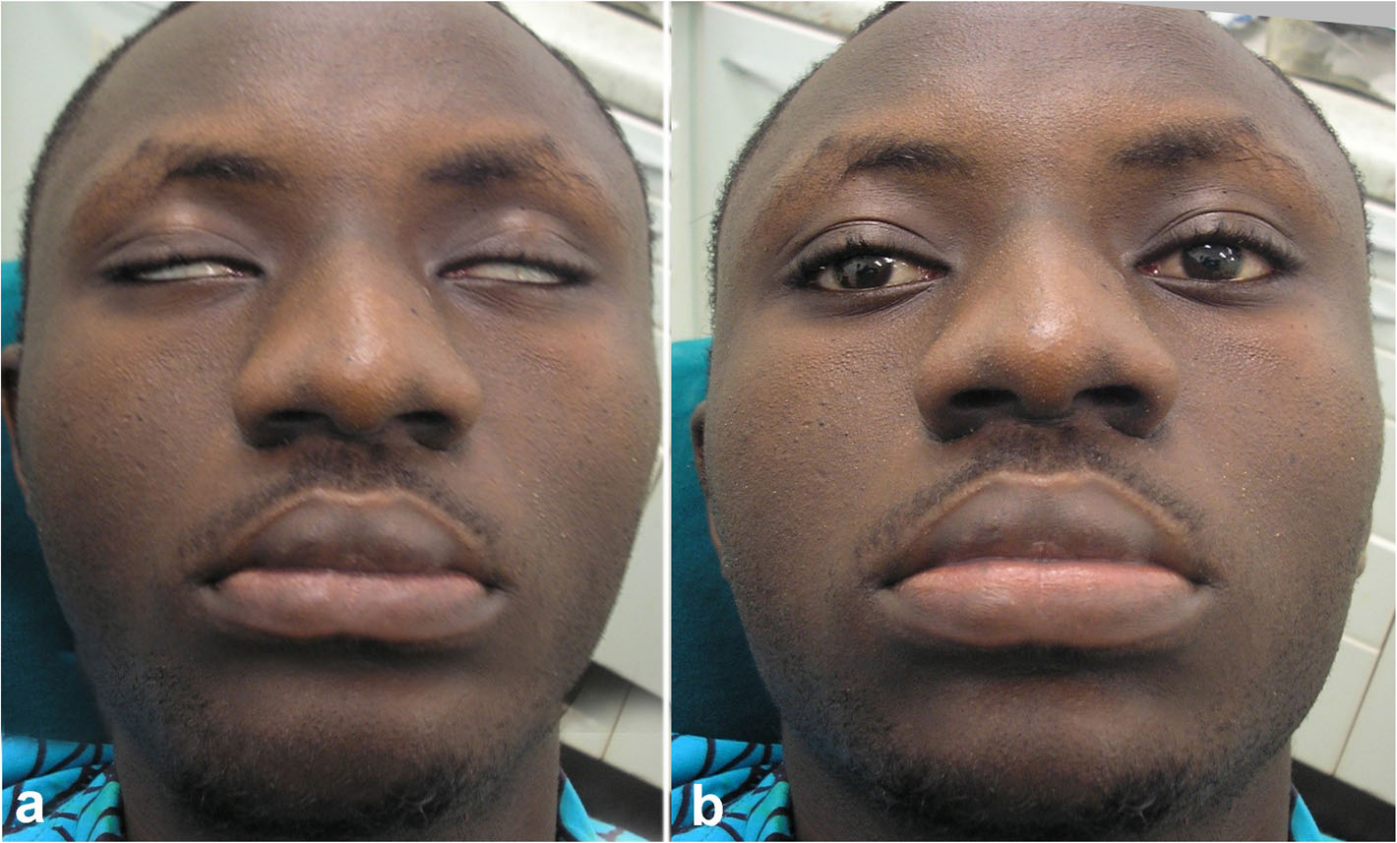
Oculogyric crisis in a patient who exhibited paroxysmal, conjugate, tonic, ocular deviations caused by sustained contractions of the ocular muscles (**a**). The patient without symptoms following treatment (**b**)

Just after initiation of treatment, the patient complained of double vision, involuntary upward eye deviation, and inability to close both eyes (Fig. 1a). The patient did not have any significant medical history. On physical examination, no signs of dyskinesia with abnormal, uncontrollable, involuntary movements were found. On neurological examination, the patient was fully alert and cooperative. Both eyes exhibited conjugate upward deviation, and the patient was unable to fully close both eyes (oculogyric crisis). The patient exhibited difficulty in bringing his eyes back to the primary position and could not sustain the position; the eyes resumed the upward position within seconds.

Upon the examination of the eyes, the pupils were isocoric and reactive to light. Ocular motility and visual acuity could not be evaluated due to the crisis. Based on the above-mentioned findings, the patient was diagnosed with Coartem-induced oculogyric crisis. AL drug treatment was immediately stopped, and 25 mg/ml promethazine hydrochloride, Phenergan® (Sanofi, Paris, France), was administered through intramuscular (IM) route. The symptoms of oculogyric crisis significantly improved 1 h after the IM injection. The patient was discharged home on the same day with oral cetirizine (Dexcel-Pharma Ltd. Daventry, UK) 10 mg daily for 5 days. He attended a follow-up visit 1 week later, and all symptoms had completely disappeared (Fig. 1b).

## Discussion

Acute dystonic reactions (ADRs) occur commonly due to drug use in the form of extrapyramidal adverse reactions particularly involuntary strong contraction of the face, neck, and back muscles. ADRs can also be a side effect of other medications, such as antiemetic, antipsychotic, antidepressant, antiepileptic, and antimalarial medications, which produce ADRs due to dopaminergic-cholinergic imbalance in the basal ganglia [3]. ADRs are characterized by involuntary contractions in the region of face, trunk, extremities, pelvis, etc [3, 4]. AL, the mainstay antimalarial drug, may cause ADRs in patients at any age, even at therapeutic dosages. The incidence of Coartem-induced oculogyric crisis has not been reported in the literature, whereas other antimalarial drugs such as artesunate/amodiaquine were reported to cause ADRs in children [3].

**Fig. 2:**
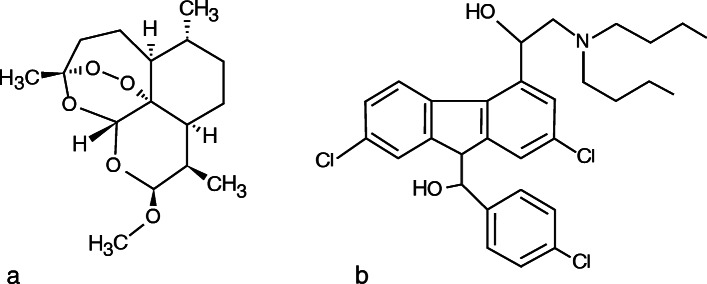
Chemical structure of Coartem. Artemether has the empirical formula of C_16_H_26_O_5_ (**a**). C_46_H_58_Cl_3_NO_6_ for Lumefantrine (**b**)

AL is thought to be highly effective in treating uncomplicated malaria in children and the frequency of associated side effects is not higher that other available artemisinin-based combination therapies [5]. Ghana is an endemic for malaria, where in 2013, 44% of all outpatient clinic visits and 22.3% of all under-five death were associated with malaria [6]. Artemisinin is highly effective in clearing the biomass of *Plasmodium* within short time and prevents the maturation of the gametocytes by its partner drug lumefantrine. Lumefantrine is an aryl amino-alcohol in the same general group as mefloquine and halofantrine, offering the maximum dual performance of AL (Fig. 2) [7]. Therefore, the aim of this case report is bring to fore the association of AL as a cause of drug-induced oculogyric crisis.

Oculogyric crisis is defined as rare nonlife-threatening neurological manifestation that causes spasmodic movements of the eyeballs into a fixed position that may last from seconds to hours [3, 8]. The spasms may be followed by emotional lability, such as restlessness, compulsive thinking, anxiety, and sensations of increased brightness or visual distortions [6]. Although oculogyric crisis is mostly drug-induced, it can be seen in hereditary and sporadic movement disorders and disorders related to focal brain lesions. Oculogyric crisis must be excluded from versive seizures, eye movement tics, paroxysmal tonic upgaze syndrome, and retinal disease. Versive seizures are phenomenologically similar to oculogyric crisis, yet they are fundamentally different [3, 9]. Wyllie et al. reported that versive seizures can mimic oculogyric crisis, but they are associated with an alteration of consciousness and should be excluded with an electroencephalogram [9]. Paroxysmal tonic upward gaze is characterized by episodes of sustained conjugate upward deviation of the eyes and can be differentiated from oculogyric crisis by the presence of neck flexion and concomitant episodic ataxia [10].

Considering the important key points including the medication history and the clinical findings of the patient, a diagnosis of AL (Coartem)-induced oculogyric crisis was made. When a patient exhibits the symptoms of a neurological disorder, such as oculogyric crisis, with conscious state and normal vital signs, special attention should be given to recently administered medications. The management of drug-induced oculogyric crisis includes discontinuation or, if not possible, reducing the dose of the opposing agent. The mainstay treatment for oculogyric crisis is anticholinergics [6]. In our case, the patient’s symptoms improved within one hour after IM injection of promethazine hydrochloride.

## Conclusions

A literature search demonstrated no published articles on Coartem-induced oculogyric crisis in Ghana, where malaria is endemic. When a patient exhibits a neurological disorder, such as oculogyric crisis, with normal conscious state and normal vital signs, special attention should be given to obtaining a history of recently administered medications. Therefore, clinicians should recognize adverse reactions to drugs based on a thorough patient history and examination and report such cases.

## Data Availability

Data sharing is not applicable to this article as no data sets were generated or analyzed during the current study.
